# Clinical characteristics of *Mycoplasma pneumoniae* compared to *Streptococcus pneumoniae* in hospitalized adults with community-acquired pneumonia- a prospective study

**DOI:** 10.1186/s12879-025-11811-8

**Published:** 2025-10-13

**Authors:** Karin Hansen, Lisa Wasserstrom, Jonas Ahl, Anna C. Nilsson, Kristian Riesbeck

**Affiliations:** 1 Clinical Microbiology, Department of Translational Medicine, Faculty of Medicine, Lund University, Malmö, Sweden; 2 Infectious Diseases, Department of Translational Medicine, Faculty of Medicine, Lund University, Malmö, Sweden; 3 Department of Clinical Microbiology, Infection Control and Prevention, Skåne University Hospital, Lund, Sweden

**Keywords:** Adults, CAP, *Mycoplasma pneumoniae*, Pneumonia, Streptococcus pneumoniae

## Abstract

**Background:**

*Streptococcus pneumoniae* is the primary cause of hospitalized community-acquired pneumonia (CAP). *Mycoplasma pneumoniae*, though typically causing mild respiratory disease, can lead to pneumonia requiring hospitalization in adults. This study compared patients hospitalized with CAP caused by *M. pneumoniae* or *S. pneumoniae* aiming at increasing the understanding of when to sample for and empirically treat *M. pneumoniae*.

**Methods:**

From 2016 to 2018, 518 adults hospitalized with CAP were prospectively and consecutively enrolled. Medical history, symptoms, radiographic, and laboratory data were recorded. Naso- and oropharyngeal swabs were collected for PCR detection of *M. pneumoniae* and other respiratory pathogens, while urine samples were analysed with two pneumococcal urinary antigens.

**Results:**

A total of 32 patients with *M. pneumoniae* and 126 patients with *S. pneumoniae* were identified. Patients with *M. pneumoniae* were significantly younger than those with pneumococcal CAP with a median age 39 versus 70 years. *Mycoplasma pneumoniae* accounted for only 6% of CAP cases across all ages, but for 33% of cases in patients < 50 years. *Mycoplasma pneumoniae* patients had a longer duration of symptoms and were more often prescribed antibiotics prior to hospital admission. Neither symptoms nor chest imaging alone could distinguish between *M. pneumoniae* and *S. pneumoniae*. Although inflammatory markers such as CRP and leukocyte counts were significantly lower in *M. pneumoniae* CAP, the median CRP value was still elevated at 178 mg/L. Viral co-detection occurred in 14% of *M. pneumoniae* patients, compared to 42% of those with *S. pneumoniae.*

**Conclusion:**

Symptoms and radiological findings could not distinguish between *M. pneumoniae* and *S. pneumoniae*. However, in hospitalized patients, particularly in younger individuals or those with antibiotic failure, liberal testing and treatment for *M. pneumoniae* is recommended.

## Introduction

Community-acquired pneumonia (CAP) is a common cause of hospitalization in adults. *Streptococcus pneumoniae* has historically been recognized as the predominate etiological agent, though the incidence of *Haemophilus influenzae* has notably increased [1–3]. *Mycoplasma pneumoniae* is known for causing epidemic outbreaks with a periodicity of 4 to 6 years [4]. It is generally associated with mild respiratory illness, primarily affecting children over 5 years [5], but it can also lead to severe disease across all age groups [6].

During endemic periods, *M. pneumoniae* is estimated to account for 4 to 8% of all bacterial pneumonia cases, rising to 20 to 40% during epidemics, with the majority being out-patients. In etiological studies, the proportion of hospitalized adults with CAP attributed to *M. pneumoniae* varies between 2 and 6% [1, 7–9]. Regarding the empirical treatment of hospitalized CAP, Swedish guidelines recommend that the initial antibiotic treatment primarily targets *S. pneumoniae*, with additional antimicrobial coverage for atypical pathogens warranted only in cases of severe pneumonia or when atypical agents are suspected based on clinical or epidemiological grounds [10]. This study is a sub-analysis of the “Etiology of CAP in Sweden” (ECAPS) study [11]. Its aim is to describe the clinical features of CAP caused by *M. pneumoniae* in comparison to pneumococcal CAP, to improve clinical knowledge and support in distinguishing between these two pathogens in an emergency room setting.

## Materials and methods

The study was conducted at Skåne University Hospital in Malmö, Sweden during September 2016 to September 2018. Consecutive hospitalized patients above 18 years of age with at least two predefined symptoms of respiratory infection and radiological finding consistent with pneumonia were screened for inclusion and prospectively enrolled. Exclusion criteria included previous hospitalization within the last 30 days. The inclusion and exclusion criteria have been previously described in detail [11].

### Ethical approval

This study was approved by the Lund Regional Ethics Committee (Nos. 2016/220 and 2016/340) and written informed consent was signed by participants or next of kin prior to inclusion.

### Data collection

Information was collected on medical history, clinical presentation, demographic variables and duration of hospital stay. Pneumonia severity was assessed using CRB-65 and pneumonia severity index (PSI). Admission and maximum values of C-reactive protein (CRP) and white cell blood count (WBC) were collected. A chart review was made for 30-day case fatality rate (CFR) as well as 30-day readmission. The chest images were interpreted by a certified clinical radiologist and categorized into different types of infiltrates based on the statements.

### Microbial detection

All microbial sampling results conducted on clinical grounds and analysed at Clinical Microbiology, Laboratory Medicine Skåne, which is accredited according to the ISO 15,189 standard, were recorded.

Per protocol testing included urine specimens tested with two principally different antigen detecting methods; BinaxNOW S. pneumoniae^®^(Abbott Diagnostics, Scarborough, ME), and UAD1 and 2; limit assays that uses Luminex technology for detection of a total of 24 specific pneumococcal serotypes [12, 13].

Flocked swab samples were taken from oro- and nasopharynx and stored at −80 °C at Clinical Microbiology. Analyses were made retrospectively using PCR for 14 respiratory viruses (influenza A H1N1, influenza A H3N2, influenza B, enterovirus, rhinovirus, parechovirus, adenovirus, human metapneumovirus [hMPV], and coronaviruses [OC43, NL63, 229E]), parainfluenza virus 1 to 3 and respiratory syncytial virus (RSV) A/B) and for 6 bacterial pathogens (*S. pneumoniae*,* H. influenzae*, *Bordetella pertussis*/*parapertussis*, *Chlamydophila pneumoniae*, and *Mycoplasma pneumoniae*). Analyses were done as previously described [14]. The definition of *M. pneumoniae* CAP was a positive PCR in at least one test; either oro- or nasopharynx in routine- or per- protocol sampling. *Streptococcus pneumoniae* CAP was defined as a positive blood or lower respiratory culture and/or positive BinaxNOW S. pneumoniae^®^ or UAD. Patients who were only positive for pneumococci in a nasopharyngeal culture and/or PCR-positive in oro- or nasopharyngeal samples were excluded from this analysis since it may represent colonization in contrast to *M. pneumoniae* which is rarely detected in asymptomatic adults in the upper respiratory tract [15–17].

### Statistical analysis

The clinical characteristics are described using numbers and percentages for categorical data, median and interquartile range [IQR] for continuous variables. Missing data are presented in the tables. The subgroups were compared with χ^2^ -test and Fishers exact test for categorical data and independent t-test or Mann-Whitney U-test for continuous variables when applicable, *p*-values < 0.05 were considered significant. Statistical analyses were made with IBM SPSS version 29.0.2.0.

## Results

**Fig. 1:**
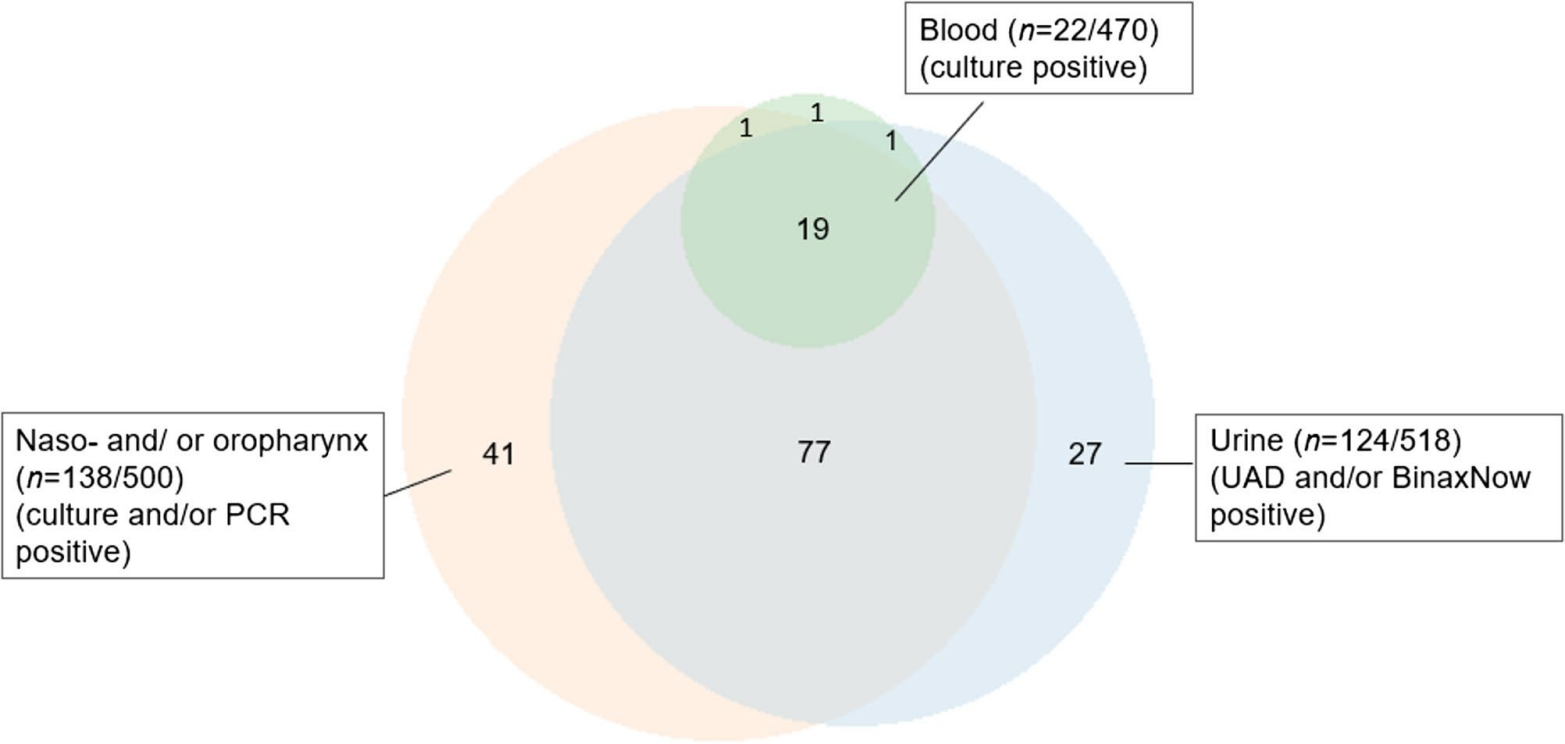
Samples analysed for *S. pneumoniae* in the present study. Lower respiratory cultures were positive in 2/42 patients, both positive in other samples and are not presented in the figure. In the naso-and oropharyngeal samples 136 were PCR-positive and 21 culture positive

Of the 518 patients included in the ECAPS cohort [11], *M. pneumoniae* and *S. pneumoniae* were detected in 33 and 168 cases, respectively. One patient tested positive for both pathogens, and 41 patients were positive only for *S. pneumoniae* by PCR in an upper respiratory sample, resulting in 32 and 126 patients included in the final analysis for *M. pneumoniae* and *S. pneumoniae*, respectively. Twenty-two patients had a positive blood culture for *S. pneumoniae*. The results of the different diagnostic tests for *S. pneumoniae* are presented in Fig. 1.

### Clinical characteristics

Patients with *M. pneumoniae* were significantly younger and healthier than those with pneumococcal CAP, with a mean age of 37 years compared to 68 years. Only 4 out of 32 patients with *M. pneumoniae* (13%) had one or more comorbidities, in contrast to the *S. pneumoniae* group, where 102 out of 126 patients with *S. pneumoniae* (81%) had underlying conditions (*p* < 0.001) (Table 1). At the time of admission, both groups presented with similar symptoms, including fever, cough, malaise and dyspnoea being the most common symptoms. Pleuritic pain was significantly more common in patients with *M. pneumoniae* (75%) compared to 41% of patients with *S. pneumoniae (p =* 0.002).

**Table 1:** Patient demography and disease severity

	*M. pneumoniae*	*S. pneumoniae*	*p*-value	All CAP	*p*-value^1^
Age, median [IQR]	36.0 [27–43]	70.0 [58–81]	< 0.001	73 (60–82)	< 0.001
Female sex (%)	15/32 (46.9)	54/126 (42.9)	0.682	236/518 (45.6)	0.877
Contact with child < 5 years last 2 weeks	11/28 (39.3)	42/126 (33.3)	0.154	140/485 (30.1)	0.834
Co-morbidities					
Smoker current (%)	6/32 (18.2)	33/126 (26.2)	0.383	97/518 (18.8)	0.999
No comorbidities	28/32 (87.5)	24/126 (19.0)	< 0.001	117/518 (22.6)	< 0.001
COPD (%)	2/32 (6.1)	42/125 (33.3)	0.002	143/514 (29.1)	0.002
Asthma (%)	1/32 (3.0)	11/126 (8.7)	0.462	47/518 (9.1)	0.334
Congestive heart failure (%)	1/32 (3.1)	18/126 (14.3)	0.126	95/518 (18.3)	0.017
Coronary artery disease (%)	1/32 (3.1)	37/126 (29.4)	< 0.001	135/518 (26.1)	0.001
Diabetes mellitus (%)	1/32 (3.1)	18/136 (14.3)	0.126	87/518 (17.7)	0.028
Immunosuppressive therapy (%)	0/32 (0)	16/126 (12.7)	0.043	65/516 (13.4)	0.024
Chronic kidney disease (%)	1/32 (3.1)	9/126 (7.1)	0.688	47/517 (9.5)	0.344
Immunodeficiency (%)^2^	0/32 (0)	7/126 (0)	0.346	27/518 (5.2)	0.394
Cancer solid tumour (%)	0/32 (0)	33/126 (26.2)	0.001	106/516 (21)	0.030
Disease Severity					
CRB-65 2–3^3^(%)	0/32 (0)	27/126 (21.4)	0.003	96/518 (18.5)	0.005
PSI- grade IV-V (%)	1/32 (3.1)	68/126 (54.0)	< 0.001	262/518 (50.5)	< 0.001
Oxygen/NIV/HFNC (%)	23/32 (71.9)^4^	76/126 (60.3)^5^	0.227	-	-
Oxygen L/min, median [IQR]	3.8 [2.0–6.0]	3.0 [2.0–4.0]	0.017	-	-
ICU-admission (%)	0/32 (0)	2/126 (1.6)	1.000	9/517 (1.7)	1.000
Length of stay, median [IQR]	4.0 [3.0–5.0]	4.0 [3.0–7.0]	0.085	5.0 [3.0–8.0]	0.005
Readmission < 30 days (%)	1/32 (3.0)	14/126 (11.1)	0.308	79/518 (15.3)	0.044
Case-fatality rate < 30 days (%)	0/32 (0.0)	4/126 (3.1)	0.583	18/518 (3.5)	0.617

^1^ *Mycoplasma pneumoniae* compared to All-CAP

^2^ Including HIV, AIDS, organ transplant and hematologic malignancy

^3^ In the whole cohort CRB-65 4 points (*n* = 0), 3 points (*n* = 14). *S. pneumoniae* 3 points (*n* = 1)

^4^ Patients with HFNC (*n* = 2), 0 patients with NIV

^5^ Patients with HFNC (*n* = 6), 1 person with NIV

**Fig. 2:**
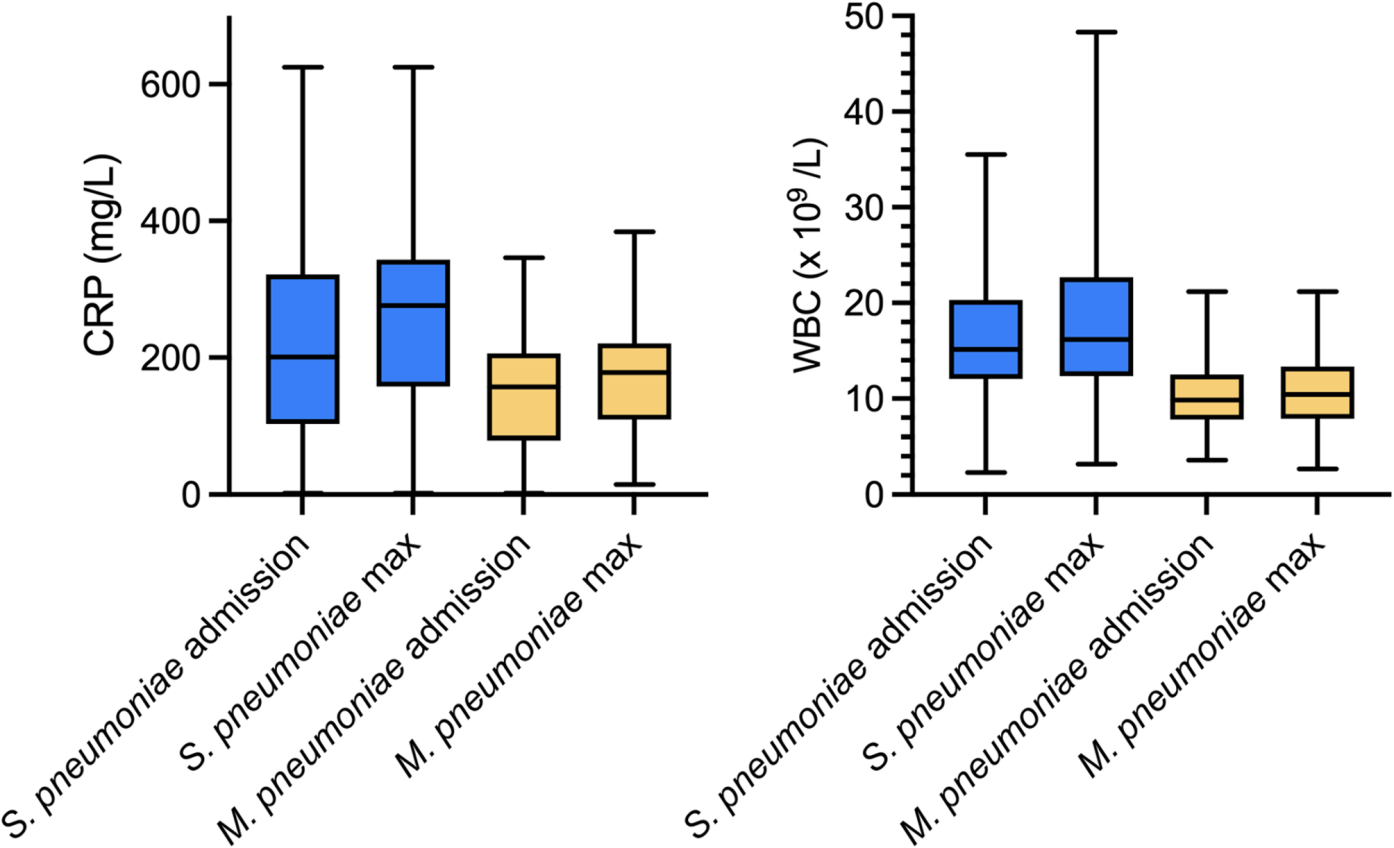
Comparison of C-reactive protein levels (CRP) and white blood cell count (WBC) for *S. pneumoniae* and *M. pneumoniae*. Reference values for WBC are 3.5–8.8 × 10^9^/L

The frequency of pleural effusion detected by X-ray, on the other hand, did not differ significantly between the groups with 6/32 (19%) of *M. pneumoniae* patients and 38/126 (30%) of *S. pneumoniae* patients. The radiological findings were predominantly unilateral in both groups, and there was no significant difference in the frequency of bilateral findings. In both patient groups, the characterisation of infiltrates varied, patchy opacities were the only type significantly more observed in *M*. *pneumoniae* (*p =* 0.032), yet they were still only seen in 4/32 in this group (Table 1). Finally, inflammatory laboratory parameters were significantly higher in pneumococcal CAP. The median maximum WBC counts were 10 × 10^9^/L in *M. pneumoniae* CAP and 16 × 10^9^/L in *S. pneumoniae* CAP (*p* < 0.001). The maximum CRP level was also significantly lower in *M. pneumoniae* patients compared to patients with *S. pneumoniae*, 178 mg/L and 263 mg/L respectively (*p* < 0.001), but with a considerable overlap (Fig. 2).

### Disease severity

Patients with pneumococcal CAP had both significantly higher CRB-scores and PSI-grades (*p <* 0.001). Among the patients with pneumococcal CAP, 83 out of 126 (69%) received supplemental oxygenation. Within this group, 7 patients required high-flow nasal cannula (HFNC)-therapy, one needed non-invasive ventilation (NIV), and another patient underwent invasive mechanical ventilation. Supplemental oxygen was needed in 25 out of 32 patients with *M. pneumoniae* (78%), among these patients two individuals were treated with HFNC therapy, but no patients required mechanical ventilation. The mean oxygen flow rate was higher in patients with *M. pneumoniae* (4.7 L/min) compared to those with *S. pneumoniae* (3.2 L/min; *p =* 0.017*)*. The overall mortality rate was low, with no patients infected with *M. pneumoniae* having died within 30 days, compared to 4 patients (3%) with pneumococcal CAP (Table 2).

**Table 2:** Clinical characteristics

	* M. pneumoniae*	*S. pneumoniae*	*p*-value	All CAP	*p*-value^1^
Symptoms					
Symptom duration median [IQR]	7.0 [5.0–11.8.0.8]	3.0 [1.0–7.0]	0.004	4.0 [2.0–7.0]	0.043
Fever (%)	32/32 (100)	113/126 (89.7)	0.058	438/518 (84.6)	0.013
Chills (%)	28/32 (87.5)	89/126 (70.6)	0.052	310/517 (59.8)	0.004
Pleuritic pain (%)	23/32 (71.9)	52/126 (41.3)	0.002	203/518 (39.2)	< 0.001
Cough (%)	31/32 (96.9)	110/126 (87.3)	0.119	439/517 (84.7)	0.143
Sputum (%)	20/32 (62.5)	78/126 (61.9)	0.951	311/518 (60.0)	0.903
Dyspnoea (%)	30/32(93.8)	98/126 (77.8)	0.040	397/518 (76.6)	0.018
Tachypnoea (%)	21/32 (65.6)	84/126 (66.7)	0.911	304/518 (58.7)	0.411
Malaise (%)	31/32 (96.9)	107/126 (84.9)	0.069	453/518 (87.5)	0.097
Abnormal lung auscultation (%)	20/32 (62.5)	99/126 (78.6)	0.060	382/517 (73.6)	0.307
CRP at admission (mg/L), median [IQR]	157 (79–206)	201 [103–321]	< 0.001	132 (58–252)	0.532
WBC at admission median (x10^9^/L) [IQR]	9.85 [7.8–12.5]	15.2 [12.1–20.3]	< 0.001	-	-
Prior antibiotic treatment^2^	17/33 (51.5)	14/126 (11.1)	< 0.001	88/484 (18.1)	< 0.001
Radiological findings^3^					
Pleuritic effusion (%)	6/32 (18.8)	38/126 (30.2)	0.199	-	-
Bilateral abnormalities (%)	13/32 (40.6)	34/126 (27.0)	0.132	-	-
Lobar consolidation (%)	20/32 (62.5)	74/126 (58.7)	0.698	-	-
Interstitial opacities (%)	3/32 (9.4)	9/126 (7.1)	0.710	-	-
Vague opacities (%)	2/32 (6.3)	24/126 (19.0)	0.109	-	-
Patchy opacities (%)	4/32 (12.5)	3/126 (2.3)	0.032	-	-
Small opacities (%)	3/32 (9.4)	16/126 (12.7)	0.767	-	-

^1^ *Mycoplasma pneumoniae* compared to All-CAP

^2^ Within 2 weeks prior to admission

^3^ The majority had a chest x-ray, 31% of *M. pneumoniae* patients and 9% of *S. pneumoniae* patients had a CT

### Antimicrobial treatment

Effective antimicrobial treatment for *M. pneumoniae* were defined as either a tetracycline, macrolide or a fluoroquinolone. In Table 3, prescribed antibiotics are listed. 50% of the patients with *M. pneumoniae* had received antibiotics within the last two weeks prior to admission, the majority was administered penicillin V, which is in line with the Swedish national guidelines [10]. Two patients had been prescribed antimicrobials targeting *M. pneumoniae*; one of them had taken two tablets of a fluoroquinolone but had been vomiting, the other patient was treated with doxycycline for 5 days prior to hospitalization but was also taking iron supplements known to lower doxycycline concentrations with as much as 90% [18]. In contrast, only 11% of the patients admitted with pneumococcal CAP had been prescribed prior antibiotics.

**Table 3:** Antibiotic treatments used in the present study

	*M. pneumoniae *(*n* = 32)				*S. pneumoniae* (*n* = 126)			
*Antibiotics n* (%)	*Before admission*^*3*^	*Day 1*	*Day 3*	*Last antibiotic*	*Before admission*^3^	*Day 1*	*Day 3*	*Last antibiotic*
Cefotaxime	0	5 (15.6)	2 (6.3)	0	0	62 (51.6)	35 (27.8)	6 (4.8)
Benzylpenicillin	0	9 (28.1)	3 (9.4)	0	0	41 (32.5)	42 (33.3)	4 (3.2)
Cefotaxime + M/FQ/D^1^	0	3 (9.4)	2 (6.3)	0	0	5 (4.0)	2 (1.6)	0
Benzylpenicillin + M/FQ/D^1^	0	10 (31.2)	4 (12.5)	0	0	4 (3.2)	2 (1.6)	0
Piperacillin-tazobactam	0	0	0	0	0	4 (3.2)	1 (0.8)	0
Carbapenem	0	0	0	0	0	1 (0.8)	0	1 (0.8)
Penicillin V	13 (40.6)	0	1 (3.1)	2 6.3)	7 (5.6)	1 (0.8)	8 (6.3)	31 (24.6)
Doxycycline	1 (3.1)	0	4 (12.5)	7 (21.9)	4 (3.2)	1 (0.8)	5 (4.0)	5 (4.0)
Macrolide	0	4 (12.5)	13 (40.6)	20 (62.5)	0	0	2 (1.6)	5 (4.0)
Fluoroquinolone	1 (3.1)	0	2 (6.3)	1 (3.1)	1 (0.8)	0	3 (2.3)	7 (5.6)
Amoxicillin	2	0	1 (3.1)	2 (6.3)	0	0	23 (18.2)	57 (45.2)
Amoxicillin-clavulanic acid	0	0	0	0	0	0	0	2 (1.6)
Other^2^	1 (3.1)	1 (3.1)	0	0	2 (1.6)	7 (5.6)	2 (1.6)	8 (6.3)
No antibiotic	16	0	0	0	112 (88.9)	0	1 (0.8)	1 (0.8)

^1^ M/FQ/D = Macrolide/Fluoroquinolone/Doxycycline

^2^ Includes Clindamycin alone or with other antibiotics and Cefotaxime and other antibiotics, i.e., Metronidazole. Two patients had Nitrofurantoin before admission

^3^ Two patients had 2 different antibiotics before admission: one patient Penicillin and then Amoxicillin, another Penicillin followed by Doxycycline

All patients with pneumococcal CAP were empirically prescribed antibiotics effective against *S. pneumoniae* at admission, most commonly a ß-lactam, either alone or in combination with another antimicrobial. Among the *M. pneumoniae* patients, 16 out of 32 received effective empirical treatment, either alone or in combination with a ß-lactam, while the remaining 14 patients were treated with a ß-lactam antibiotic alone. On the third day of hospitalization, 5 patients remained on only ß-lactam, and the rest had added or switched to single treatment with a *M. pneumoniae*-active antimicrobial agent. Four patients with *M. pneumoniae* were discharged with penicillin V or amoxicillin; three of them required oxygen but rapidly improved (Table 3). Two were not clinically tested for *M. pneumoniae*, one tested negative in routine testing but positive in the per protocol oropharyngeal sample, and the other was positive in the routine testing, with results arriving 3 days after discharge. This patient was in a worse clinical state and readmitted for 5 days to receive adequate treatment. This was the only *M. pneumoniae* patient readmitted within 30 days.

### Co-detections and seasonality

**Fig. 3:**
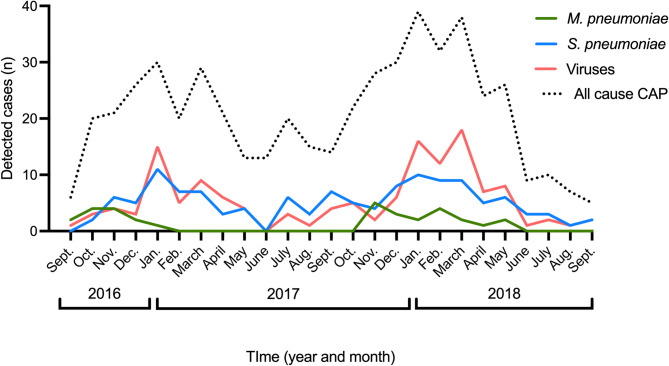
Monthly detection of *M. pneumoniae*,* S. pneumoniae*, and respiratory viruses during the study period. Viruses include influenza A/B, enterovirus, rhinovirus, parechovirus, adenovirus, human metapneumovirus and coronaviruses [OC43, NL63, 229E], parainfluenza virus 1 to 3 and respiratory syncytial virus A/B

Simultaneous detection of viruses was found in 4 out of 32 cases (14%) with *M. pneumoniae*, compared to 42% of cases suffering from pneumococcal CAP (*p =* 0.006). *Mycoplasma pneumoniae* infections showed two peaks during the study period, with the highest detection rates between October 2016 and December 2016, and from December 2017 to May 2018, with no detected cases in between. In contrast, *S. pneumoniae* exhibited three peaks which followed, and may have been related to, the seasonality of detected viruses (Fig. 3).

## Discussion

In Northern Europe, the empirical antibiotic strategy for treating non-severe CAP in hospitalized patients is a ß-lactam antibiotic, with the addition of a macrolide or fluoroquinolone reserved only for severe cases or when there is a clinical suspicion of atypical pneumonia [10, 19]. Our objective was to determine when to suspect the most common atypical pneumonia: *Mycoplasma pneumoniae* CAP by comparing it to pneumococcal CAP. Most importantly, *M. pneumoniae* was overrepresented in patients below 50 years of age and more frequently associated with experienced antibiotic failure before hospital admission. Clinical symptoms and radiographic findings were insufficient to discriminate between the two pathogens, but inflammatory laboratory results, especially WBC count, could provide guidance.

The strengths of our study are its prospective design and comprehensive testing, which made missed *M. pneumoniae* cases unlikely in the cohort. One limitation, however, was the difficulty in including critically ill patients, which may have led to their underrepresentation. In addition, the requirement to provide a urine sample as an inclusion criterion excluded anuric patients with acute kidney injury or end-stage renal disease. We chose to exclude cases that were only positive for *S. pneumoniae* in upper respiratory tract samples due to the difficulty to discriminate between colonization and infection. This may have led to an underrepresentation of *S. pneumoniae* cases. Finally, despite extensive testing, the number of detected *M. pneumoniae* cases was low, suggesting an endemic setting in which selecting patients for sampling from a resource-saving perspective can be more challenging.

*Streptococcus pneumoniae* was as expected, a far more common finding, with *M. pneumoniae* accounting for only 6% of all hospitalized CAP cases during a non-epidemic period, in line with other studies [7–9]. The most prominent difference between the two pathogens was the age disparity, with a median age of 36 years for *M. pneumoniae* and 70 years for pneumococcal CAP. Similar median ages of 39–43 years were reported in four European studies on *M. pneumoniae* CAP [20–23]. However, Dumke et al. included out-patients, and Metsälä et al. included children, both of which would lower the median age [21, 24]. In addition, three of these studies were retrospective, potentially leading to missed cases. In our cohort, 97% of all CAP patients were tested for *M*. *pneumoniae* and only two cases (6%) were detected in patients over 65 years, one of whom improved despite inadequate treatment. A prospective Japanese study using serology found that 15% of patients with detected *M. pneumoniae* were over 60 years old, while a retrospective Israeli study on PCR-positive patients reported that 17% of patients were over 65 years old [6, 25]. Both studies covered long time periods (6–15 years), likely including epidemic years, which could explain these findings. Further, relying on serology has limitations especially if only a single measurement of IgG or IgM is made, in the cited study however, both acute and convalescent sera were taken.

Fever, malaise, cough and dyspnoea were the most common symptoms of *M. pneumoniae* CAP. Dyspnoea and pleuritic pain were the only symptoms significantly more common in *M. pneumoniae* patients than in those with pneumococcal CAP, and also more common compared to all-cause CAP, interestingly since pleuritic pain has been considered a more specific sign of pneumococcal CAP [26]. Laboratory findings showed differences between the two pathogens with significantly lower CRP and WBC levels for *M. pneumoniae* compared to CAP caused by *S. pneumoniae.* Despite this, the CRP level in *M. pneumoniae* cases was still elevated with a median value of 178 mg/L at admission, similar to findings from a retrospective study on hospitalized CAP patients [22], suggesting that CRP-levels alone have limited value for diagnosing *M. pneumoniae*. In 75% of *M. pneumoniae* cases, WBC levels at admission were below 12.5 × 10^9^/L, whereas in 75% of pneumococcal CAP, WBC levels exceeded 12.1 × 10^9^/L at admission.

Radiographic findings in our cohort of hospitalized patients suggest that *M. pneumoniae* can present with a variety of appearances. Unilateral lobar consolidation was the most common finding, while patchy, interstitial, and vague opacities as well as bilateral abnormalities, were also observed. Studies focusing on radiology have found that bronchial wall thickening and centrilobular nodes are more indicative of *M. pneumoniae* than pneumococcal pneumonia [27]. However, this was not noted in our study as chest X-rays, rather than computed tomography, were used in most cases, further, a large retrospective French study also found that the radiographic imaging in *M. pneumoniae* CAP was highly polymorphic [23]. The most common radiographic finding in our study for pneumococcal CAP was unilateral consolidation; however, bilateral findings, interstitial patterns, and vague or small opacities were also observed, as previously described [28].

In this study, *M*. *pneumoniae* patients had significantly lower CRB scores and PSI grades. The majority of hospitalized patients with *M*. *pneumoniae* CAP required oxygen, and those in the *M. pneumoniae* group were treated with significantly higher oxygen levels than patients with pneumococcal pneumonia. Few patients in either group underwent HFNC-therapy, likely due to data collection occurring before the COVID-19 pandemic, after which HFNC usage has increased. It is expected that this therapy would now be more prevalent in both patient groups.

Only few patients with CAP caused by *S. pneumoniae* underwent mechanical ventilation or non-invasive ventilation, and none of the *M. pneumoniae* patients did. Previous studies have shown varying results regarding disease severity: three studies reported no mortality in analogy with our results [20, 21, 27], while two studies found two cases of death [22, 29]. Another investigation showed 6% mortality, with 16% of patients admitted to the ICU and 9% requiring mechanical ventilation [6]. In a retrospective Swedish study involving 388 patients, 8% of PCR-positive *M. pneumoniae* patients were admitted to the ICU, and one person died [22], and in a French recent observational report more than 30% were admitted to the ICU, and a total in hospital mortality of 2%, it was however noted that HFNC-treatment were only administered in the ICU which likely explained the high ICU-admission rates [23].

Patients with *M. pneumoniae* CAP had a longer duration of illness before hospitalization and were more likely to have been prescribed antibiotics within two weeks of admission. This frequent prescription of antimicrobial drugs was anticipated, as the first line treatment for CAP in Sweden for outpatients is penicillin V, which targets pneumococci but is ineffective against *M. pneumoniae*, which lacks a cell wall [10]. Viral co-detection was significantly more common in pneumococcal CAP compared to *M. pneumoniae* CAP. A study by Diaz et al. had a similar rate of 10% viral co-detections [30], suggesting that *M. pneumoniae* as an etiology is not a common cause of superinfection.

We found that during an endemic period, 27% of all hospitalized CAP patients underwent testing for *M. pneumoniae* based on clinical suspicion. When the entire cohort was tested, only two additional cases were confirmed in the previously untested group. However, three cases from routine sampling were false negative, while one patient tested positive in routine sampling but negative in the per protocol sampling, emphasizing that a test is only as reliable as the quality of the sample. The delay in effective treatment, however, did not affect the outcome. While a carrier state is not uncommon in children [31], we have previously found that *M. pneumoniae* is a rare finding in asymptomatic adults [14].

## Conclusion

Neither symptoms nor X-ray findings were sufficient to distinguish between *M. pneumoniae* and pneumococcal CAP. However, patients with *M*. *pneumoniae* infection rarely had co-morbidities, were more likely to have been prescribed antibiotics as out-patients and had a longer duration of symptoms. Patients with *M. pneumoniae* were also more than three decades younger. In a non-epidemic setting, 33% of hospitalized patients under 50 years of age tested positive for *M. pneumoniae*, suggesting that liberal testing in this age group is warranted. The strategy of empirically treating patients with ß-lactam only in non-severe CAP did not affect outcome.

## Data Availability

The datasets generated and/or analysed during the current study are not publicly available due to privacy concerns but are available from the corresponding author on reasonable request.

## Abbreviations

CAP: Community-acquired pneumonia
CFR: Case-fatality rate
CRP: C-reactive protein
ECAPS: Etiology of community-acquired pneumonia in Sweden
HFNC: High flow nasal cannula
IQR: Interquartile range
NIV: Non-invasive ventilation
PSI: Pneumonia severity index
WBC: White cell blood count
