# Correction to: Clinical characteristics of *Mycoplasma pneumoniae* compared to *Streptococcus pneumoniae* in hospitalized adults with community-acquired pneumonia- a prospective study

**DOI:** 10.1186/s12879-025-12014-x

**Published:** 2025-11-07

**Authors:** Karin Hansen, Lisa Wasserstrom, Jonas Ahl, Anna C. Nilsson, Kristian Riesbeck

**Affiliations:** 1 Clinical Microbiology, Department of Translational Medicine, Faculty of Medicine, Lund University, Malmö, Sweden; 2 Infectious Diseases, Department of Translational Medicine, Faculty of Medicine, Lund University, Malmö, Sweden; 3 Department of Clinical Microbiology, Infection Control and Prevention, Skåne University Hospital, Lund, Sweden

**Correction to: BMC Infectious Diseases (2025) 25:1292**

10.1186/s12879-025-11811-8

Following publication of the original article [1], we were notified of a few errors that occurred during the proofing stage:

In Table 1 the headlines should read “M. pneumoniae” and “S. pneumoniae” instead of “Sho S. pneumoniae”.

In Table 2 the headlines should read “M. pneumoniae” and “S. pneumoniae” instead of “Sho M. pneumoniae”.

The original article has been corrected.
